# Interim Effectiveness Estimates of 2025 Southern Hemisphere Influenza Vaccines in Preventing Influenza-Associated Outpatient and Hospitalized Illness — Eight Southern Hemisphere Countries, March–September 2025

**DOI:** 10.15585/mmwr.mm7436a3

**Published:** 2025-09-25

**Authors:** Savanah Russ, Francisco Nogareda, Annette K. Regan, Estefanía Benedetti, Marina Pasinovich, Carla Voto, Monique Chilver, Nigel Stocks, Sheena G. Sullivan, Allen C. Cheng, Christopher C. Blyth, Jenna Hassall, Walquiria Aparecida Ferreira de Almeida, Francisco José de Paula Júnior, Ana Catarina de Melo Araújo, Natalia Vergara, Paula Camila Rodríguez Ferrari, Rodrigo A. Fasce, Christian Saavedra, Elena Penayo, Silvia Gómez, Chavely Domínguez, Andrew Anglemyer, Tim Wood, Q. Sue Huang, Sibongile Walaza, Phindi Zwane, Nicole Wolter, Natalia Goñi, Jeremy Tairovich, Eduardo Silvera, Paula Couto, Jorge Jara, Rebecca J. Kondor, Eduardo Azziz-Baumgartner, Anna N. Chard

**Affiliations:** ^1^Influenza Division, National Center for Immunization and Respiratory Diseases, CDC; ^2^Epidemic Intelligence Service, CDC; ^3^Pan American Health Organization, Washington, DC; ^4^Servicio Virosis respiratorias, Laboratorio Nacional de Referencia INEI-ANLIS Carlos G Malbran, Buenos Aires, Argentina; ^5^Secretaría de Gestión Administrativa, Ministerio de Salud de la Nación, Buenos Aires, Argentina; ^6^Direccion de Epidemiologia, Ministerio de Salud de la Nación, Buenos Aires, Argentina; ^7^Discipline of General Practice, Adelaide Medical School, The University of Adelaide, Adelaide, Australia; ^8^School of Clinical Sciences, Monash University, Melbourne, Australia; ^9^Monash Infectious Diseases, Monash Health and School of Clinical Sciences, Monash University, Australia; ^10^School of Medicine and Wesfarmers Centre of Vaccines and Infectious Disease, The Kids Research Institute Australia, University of Western Australia, Perth, Australia; ^11^Department of Infectious Diseases, Perth Children’s Hospital, Perth, Australia; ^12^PathWest Laboratory Medicine WA, QEII Medical Centre, Perth, Australia; ^13^Australian Government Department of Health, Disability and Ageing, Canberra, Australia; ^14^Coordenação Geral de Vigilância da Covid-19, Influenza e Outros Vírus Respiratórios, Ministério da Saúde, Brasília, Brasil; ^15^Departamento do Programa Nacional de Imunizações, Ministério da Saúde, Brasília, Brazil; ^16^Departamento de Epidemiología, Ministerio de Salud de Chile, Santiago, Chile; ^17^Subdepartamento Enfermedades Virales, Instituto de Salud Pública de Chile, Santiago, Chile; ^18^Programa Nacional de Inmunizaciones, Ministerio de Salud de Chile, Santiago, Chile; ^19^Area de Vigilancia Especiales y Centinela, Ministerio de Salud Pública y Bienestar Social, Asunción, Paraguay; ^20^New Zealand Institute for Public Health and Forensic Science, Wellington, New Zealand; ^21^School of Public Health, Faculty of Health Sciences, University of the Witwatersrand, Johannesburg, South Africa; ^22^Centre for Respiratory Diseases and Meningitis, National Institute for Communicable Diseases of the National Health Laboratory Service, Johannesburg, South Africa; ^23^School of Pathology, Faculty of Health Sciences, University of the Witwatersrand, Johannesburg, South Africa; ^24^Departamento de Laboratorios de Salud Pública, Ministerio de Salud Pública, Montevideo, Uruguay; ^25^Area de Vigilancia en Salud de la Población, Ministerio de Salud Pública, Montevideo, Uruguay; ^26^Departamento de Vigilancia en Salud, Ministerio de Salud Pública, Montevideo, Uruguay.

**Summary**

**What is already known about this topic?**

Monitoring seasonal influenza vaccine effectiveness in Southern Hemisphere countries can guide health authorities in Northern Hemisphere countries about the potential protection provided from vaccination.

**What is added by this report?**

During the 2025 Southern Hemisphere influenza season, seasonal influenza vaccination reduced influenza-associated outpatient visits by 50.4% and hospitalization by 49.7%.

**What are the implications for public health practice?**

CDC recommends that all eligible persons aged ≥6 months receive the seasonal influenza vaccine. The 2025–26 Northern Hemisphere seasonal influenza vaccine composition is the same as that used during the 2025 Southern Hemisphere influenza season and might be similarly effective if the same viruses circulate in the coming season.

## Abstract

Seasonal influenza vaccination provides important protection from influenza illness and associated potential complications. Monitoring seasonal influenza vaccine effectiveness (VE) in Southern Hemisphere countries can apprise health authorities in Northern Hemisphere countries about the potential protection provided from vaccination. Using data from influenza-like illness (ILI) and severe acute respiratory infection (SARI) sentinel surveillance networks in eight Southern Hemisphere countries, investigators estimated interim VE against influenza-associated outpatient visits and hospitalization using a test-negative case-control study design. During March–September 2025, Australia and South Africa identified 2,122 patients with ILI; Argentina, Australia, Brazil, Chile, New Zealand, Paraguay, and Uruguay identified 42,752 patients with SARI. Overall, 21.3% of patients with ILI and 15.9% of patients with SARI were vaccinated against influenza. Adjusted VE against influenza-associated outpatient visits and hospitalization was 50.4% and 49.7%, respectively, for any influenza virus, and 45.4% and 46.1%, respectively, for influenza A viruses. Adjusted VE against hospitalization with the predominant influenza subtype, A(H1N1)pdm09, was 41.6%. These interim estimates suggest that vaccination reduced medically attended influenza-associated illness by approximately one half in eight Southern Hemisphere countries. Health authorities should prioritize vaccination of all eligible persons ≥6 months to reduce incidence of influenza disease.

## Introduction

Each year, influenza virus infections result in approximately 5 million hospitalizations and 650,000 deaths worldwide. Virus circulation tends to occur during April–September in Southern Hemisphere and October–May in Northern Hemisphere temperate countries (*1*,*2*). Influenza vaccination during campaigns targeting eligible persons, including groups at higher risk for severe influenza illness (e.g., young children, persons with comorbidities, and older adults), contributes to the reduction in influenza-associated morbidity and mortality worldwide (*3*,*4*). Sentinel surveillance systems facilitate systematic monitoring of seasonal influenza vaccine effectiveness (VE), which provides information to guide public health messaging and influenza vaccine composition deliberations each season (*5*). This analysis used data from influenza-like illness (ILI) and severe acute respiratory infection (SARI) sentinel surveillance networks in eight Southern Hemisphere countries to estimate interim VE against influenza-associated outpatient visits and hospitalization.

## Methods

### Data Sources

Patients with ILI, who were examined in an outpatient setting, and patients with SARI, who were admitted to a hospital, were identified through sentinel surveillance systems in eight countries.^†^ As part of the sentinel surveillance protocols, respiratory specimens from patients who met the ILI or SARI case definition were tested for influenza viruses by reverse-transcription–polymerase chain reaction (RT-PCR) and typed and subtyped in national reference laboratories. One country (South Africa) contributed only ILI surveillance data. Six countries contributed only SARI surveillance data: New Zealand and five countries (Argentina, Brazil,^§^ Chile, Paraguay, and Uruguay) from the Pan American Health Organization Network for the Evaluation of Vaccine Effectiveness in Latin America and the Caribbean–influenza (Red para la Evaluación de Vacunas en Latino América y el Caribe–influenza [REVELAC-i]). One country (Australia) contributed both ILI and SARI surveillance data.

ILI and SARI surveillance data during March–September 2025 were pooled across 163 general practitioner practices and across 3,157 hospitals, respectively (SupplementaryTable1). VE evaluation began on the date of the first influenza case detection after the start of the influenza vaccination campaign in each respective country.^¶^ All countries used WorldHealthOrganization(WHO)-recommended egg-based, inactivated Southern Hemisphere influenza vaccine formulations.**

### Study Design

VE against influenza-associated outpatient visits and hospitalization was estimated using a test-negative case-control design. The study population comprised patients with ILI or SARI who were vaccination-eligible in each country, based on national policy. Case-patients were those who received a positive influenza RT-PCR test result; control patients were those who received a negative influenza RT-PCR test result. Vaccination status was ascertained using national vaccination registries, medical records, or self-report. Patients who received a 2025 influenza vaccine dose ≥14 days before symptom onset were considered vaccinated; those not vaccinated before symptom onset were considered unvaccinated. Patients who were vaccinated <14 days before symptom onset or who received a concurrent positive SARS-CoV-2 RT-PCR test result were excluded (*6*).

### Data Analysis

VE was calculated by comparing the odds of influenza vaccination between case- and control patients using multivariable logistic regression. Models were adjusted for sex, age (in years, fit as a cubic spline with five knots), week of symptom onset (fit within each country as a cubic spline with five knots), and country. Overall VE was estimated among all patients eligible for the influenza vaccine (i.e., all patients aged ≥6 months) using STATA statistical software (version 17.0, StataCorp).^††^ In addition, VE was estimated among patients included in one of three mutually exclusive influenza vaccination priority groups considered high risk for severe outcomes associated with influenza infection (young children,^§§^ persons [older children and adults] with comorbidities,^¶¶^ and older adults***) as defined by national policy (SupplementaryTable1). When sufficient data were available, VE was estimated within the nationally defined influenza priority vaccination groups by including an interaction term between vaccination status and inclusion in a priority group and exponentiating the linear combination of the estimated coefficients. Analyses were stratified by influenza virus type and subtype when the expected frequency of vaccinated and unvaccinated case- and control patients in each stratum was at least five, and when the 95% CI for the adjusted odds ratio spanned <140 percentage points from lower to upper bound.

The frequency of influenza viral clades reported by study countries to the GlobalInitiativeonSharingAllInfluenzaData(GISAID) during their respective evaluation period was calculated. This activity was reviewed by CDC, deemed not research, and conducted consistent with applicable federal law and CDC policy.^†††^

## Results

### Characteristics of the Study Population

During March–September 2025, a total of 2,554 patients with ILI and 181,566 patients with SARI were identified through the included surveillance networks; among these, 432 patients with ILI and 138,814 patients with SARI were ineligible for inclusion or were excluded because influenza RT-PCR results, vaccination status, or demographic data were missing (SupplementaryTable2). Among the 2,122 included patients with ILI, 1,442 (68.0%) were from Australia and 680 (32.0%) were from South Africa; 50.3% of patients belonged to an influenza vaccination priority group considered high risk for severe outcomes associated with influenza infection. Among the 42,752 included patients with SARI, 4,499 (10.5%) were from Australia, 1,335 (3.1%) were from New Zealand, 2,028 (4.7%) were from Argentina, 28,962 (67.7%) were from Brazil, 2,286 (5.3%) were from Chile, 3,314 (7.8%) were from Paraguay, and 328 (0.8%) were from Uruguay; 85.3% of patients belonged to an influenza vaccination priority group (Table 1).

**TABLE 1:** Seasonal influenza vaccination status and influenza test results among patients with influenza-like illness and patients with severe acute respiratory infection, by selected characteristics — eight Southern Hemisphere countries,* March–September 2025

Characteristic	No. (column %)	Vaccination status, no. (row %)^†^		p-value^§^	Influenza test result, no. (row %)		p-value^§^
		Unvaccinated	Vaccinated		Negative	Positive	
**Patients with ILI, total**	**2,122**	**1,669 (78.7)**	**453 (21.3)**	**—**	**1,559 (73.5)**	**563 (26.5)**	**—**
Priority vaccination group^¶^	1,067 (50.3)	755 (70.8)	312 (29.2)	—	841 (78.8)	226 (21.2)	—
Young children	247 (11.6)	196 (79.4)	51 (20.6)	<0.001	218 (88.3)	29 (11.7)	<0.001
Persons with comorbidities	490 (23.1)	380 (77.6)	110 (22.4)		350 (71.4)	140 (28.6)	
Older adults	330 (15.6)	179 (54.2)	151 (45.8)		273 (82.7)	57 (17.3)	
Sex							
Female	1,187 (55.9)	917 (77.3)	270 (22.7)	0.076	887 (74.7)	300 (25.3)	0.14
Male	935 (44.1)	752 (80.4)	183 (19.6)		672 (71.9)	263 (28.1)	
Country							
Australia	1,442 (68.0)	1,014 (70.3)	428 (29.7)	<0.001	1,088 (75.5)	354 (24.5)	0.003
New Zealand	—	—	—		—	—	
South Africa	680 (32.0)	655 (96.3)	25 (3.7)		471 (69.3)	209 (30.7)	
REVELAC-i countries	—	—	—		—	—	
Argentina	—	—	—		—	—	
Brazil	—	—	—		—	—	
Chile	—	—	—		—	—	
Paraguay	—	—	—		—	—	
Uruguay	—	—	—		—	—	
Influenza test result							
Negative	1,559 (73.5)	1,184 (75.9)	375 (24.1)	—	1,559 (100.0)	—	—
Positive (all)	563 (26.5)	485 (86.1)	78 (13.9)		—	563 (100.0)	
Influenza A	464 (82.4)	399 (86.0)	65 (14.0)		—	464 (100.0)	
Influenza A(H1N1)pdm09	185 (39.9)	144 (77.8)	41 (22.2)		—	185 (100.0)	
Influenza A(H3N2)	211 (45.5)	205 (97.2)	6 (2.8)		—	211 (100.0)	
Influenza A (unknown subtype)	68 (14.7)	50 (73.5)	18 (26.5)		—	68 (100.0)	
Influenza B	99 (17.6)	86 (86.9)	13 (13.1)		—	99 (100.0)	
**Patients with SARI, total**	**42,752**	**35,971 (84.1)**	**6,781 (15.9)**	**—**	**24,965 (58.4)**	**17,787 (41.6)**	**—**
Priority vaccination group^¶^	36,455 (85.3)	30,089 (82.5)	6,366 (17.5)	—	21,755 (59.7)	14,700 (40.3)	—
Young children	16,426 (38.4)	13,935 (84.8)	2,491 (15.2)	<0.001	13,107 (79.8)	3,319 (20.2)	<0.001
Persons with comorbidities	7,066 (16.5)	6,286 (89.0)	780 (11.0)		3,674 (52.0)	3,392 (48.0)	
Older adults	12,963 (30.3)	9,868 (76.1)	3,095 (23.9)		4,974 (38.4)	7,989 (61.6)	
Sex							
Female	21,309 (49.8)	18,090 (84.4)	3,353 (15.6)	0.20	12,962 (60.4)	8,481 (39.6)	<0.001
Male	21,443 (50.2)	17,881 (83.9)	3,428 (16.1)		12,003 (56.3)	9,306 (43.7)	
Country							
Australia	4,499 (10.5)	3,394 (75.4)	1,105 (24.6)	<0.001	2,318 (51.5)	2,181 (48.5)	<0.001
New Zealand	1,335 (3.1)	1,077 (80.7)	258 (19.3)		894 (67.0)	441 (33.0)	
South Africa	—	—	—		—	—	
REVELAC-i countries							
Argentina	2,028 (4.7)	1,738 (85.7)	290 (14.3)	<0.001	1,459 (71.9)	569 (28.1)	
Brazil	28,962 (67.7)	24,882 (85.9)	4,080 (14.1)		15,698 (54.2)	13,264 (45.8)	
Chile	2,286 (5.3)	1,388 (60.7)	898 (39.3)		1,679 (73.4)	607 (26.6)	
Paraguay	3,314 (7.8)	3,218 (97.1)	96 (2.9)		2,680 (80.9)	634 (19.1)	
Uruguay	328 (0.8)	274 (83.5)	54 (16.5)		237 (72.3)	91 (27.7)	
Influenza test result							
Negative	24,965 (58.4)	20,617 (82.6)	4,348 (17.4)	—	24,965 (100.0)	—	—
Positive (all)**	17,787 (41.6)	15,354 (86.3)	2,433 (13.7)		—	17,787 (100.0)	
Influenza A	16,885 (94.9)	14,519 (86.0)	2,366 (14.0)		—	16,885 (100.0)	
Influenza A(H1N1)pdm09	9,914 (58.7)	8,582 (86.6)	1,332 (13.4)		—	9,914 (100.0)	
Influenza A(H3N2)	4,490 (26.6)	3,880 (86.4)	610 (13.6)		—	4,490 (100.0)	
Influenza A, unknown subtype	2,481 (14.7)	2,057 (82.9)	424 (17.1)		—	2,481 (100.0)	
Influenza B	837 (4.7)	781 (93.3)	56 (6.7)		—	837 (100.0)	

**Abbreviations:** ILI = influenza-like illness; REVELAC-i = Pan American Health Organization Network for the Evaluation of Vaccine Effectiveness in Latin America and the Caribbean–influenza (Red para la Evaluación de Vacunas en Latino América y el Caribe–influenza); SARI = severe acute respiratory infection.

* Argentina, Australia, Brazil, Chile, New Zealand, Paraguay, South Africa, and Uruguay.

^†^ Patients who received ≥1 dose of the 2025 season influenza vaccine ≥14 days before symptom onset were considered vaccinated; patients who did not receive any influenza vaccine during the 2025 season by the time of symptom onset were considered unvaccinated. Patients vaccinated 0–13 days before symptom onset or who received positive SARS-CoV-2 reverse-transcription–polymerase chain reaction test results were excluded.

^§^ Pearson’s chi-square test was used to ascertain differences in the number of persons who were vaccinated and unvaccinated or who received positive and negative influenza test results.

^¶^ Priority vaccination groups are included as mutually exclusive groups of persons considered at high risk for severe outcomes associated with influenza infection. Young children were defined as follows, by country and age: Argentina = 6 months–1 year; Australia and Uruguay = 6 months–4 years, Brazil = 6 months–5 years; Paraguay = 6 months–2 years, Chile = 6 months–10 years, and South Africa and New Zealand = not applicable (young children not included as a priority group). Older adults were defined as those aged ≥60 years (Brazil, Chile, and Paraguay) and aged ≥65 years (Argentina, Australia, New Zealand, South Africa, and Uruguay). The preexisting conditions considered by all eight countries include chronic respiratory disease (including asthma and chronic obstructive pulmonary disease), cancer, cardiovascular disease (including hypertension and stroke), diabetes and chronic renal disease, and immunocompromising conditions (including HIV/AIDS). Some conditions were considered by select countries including obesity (Argentina, Australia, Brazil, Chile, Paraguay and Uruguay), chronic neurologic diseases (Australia and New Zealand), chronic liver disease (Australia and New Zealand), tuberculosis (New Zealand and South Africa), current alcohol/drug dependency (New Zealand), chronic hematologic disorder (Australia and New Zealand), chronic metabolic disorder (Australia), and long-term aspirin therapy in children aged 5–19 years (Australia).

** Fifty-three SARI case-patients from Argentina and 12 SARI case-patients from Brazil did not have an influenza type or subtype reported.

### Case-Patient Influenza Typing and Subtyping Results

Among patients with ILI and patients with SARI, 563 (26.5%) and 17,787 (41.6%), respectively, received positive influenza RT-PCR test results. Most identified viruses were influenza A (ILI case-patients = 464; 82.4% and SARI case-patients = 16,885; 94.9%); influenza B viruses were identified among 99 (17.6%) ILI case-patients and 837 (4.7%) SARI case-patients. Among the 464 influenza A viruses identified from ILI case-patients, 211 (45.5%) were A(H3N2), 185 (39.9%) were A(H1N1)pdm09, and 68 (14.7%) were not subtyped. Among the 16,885 influenza A viruses identified from SARI case-patients, 4,490 (26.6%) were A(H3N2), 9,914 (58.7%) were A(H1N1)pdm09, and 2,481 (14.7%) were not subtyped (Figure).

**FIGURE:**
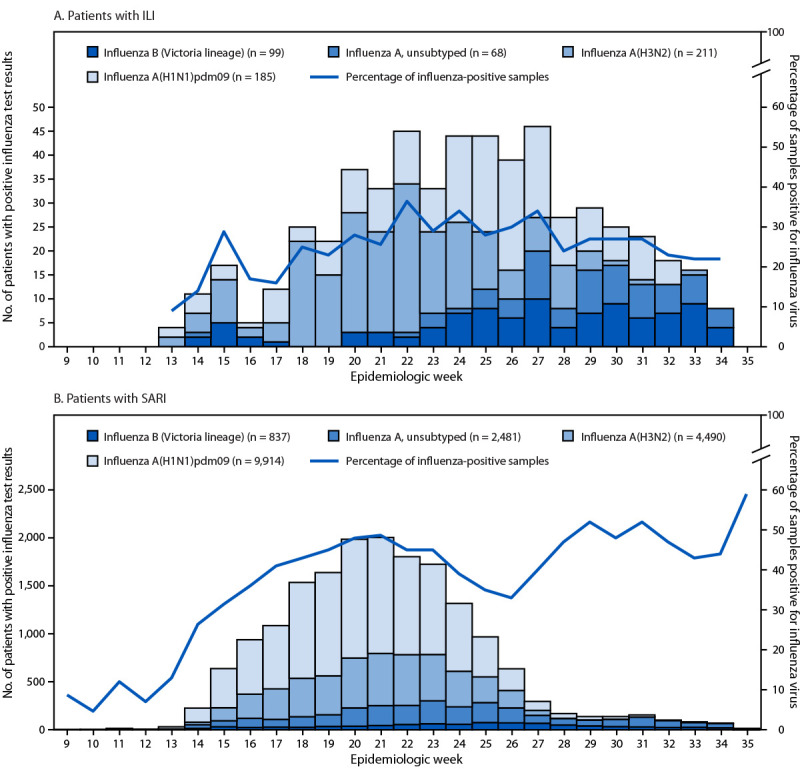
Number of patients with influenza-like illness (A)* and patients with severe acute respiratory infection^†^ (B) with positive influenza test results, by week,^§^^,^^¶^ influenza type and subtype, and percentage of all samples positive for influenza virus — eight Southern Hemisphere countries, 2025 **Abbreviations:** ILI = influenza-like illness; SARI = severe acute respiratory infection. * Patients with ILI reported from Australia (weeks 13-35) and South Africa (weeks 13-30). ^†^ Patients with SARI reported from Argentina (weeks 13-27), Australia (weeks 13-35), Brazil (weeks 14-27), Chile (weeks 9-26), New Zealand (weeks 14-34), Paraguay (weeks 14-26), and Uruguay (weeks 16-25). ^§^ ILI surveillance begins in week 13 and concludes in week 34. ^¶^ Epidemiologic week 9 started on March 1, 2025; epidemiologic week 35 ended on September 1, 2025.

### Vaccination Status of Case-Patients and Control Patients

Overall, 453 of 2,122 (21.3%) patients with ILI and 6,781 of 42,752 (15.9%) patients with SARI were vaccinated. Among the 563 ILI case-patients, 78 (13.9%) had received a 2025 seasonal influenza vaccine, compared with 375 (24.1%) of 1,559 ILI control patients (Table 1). Of the 17,787 SARI case-patients identified, 2,433 (13.7%) had received a 2025 seasonal influenza vaccine compared with 4,348 (17.4%) of 24,965 SARI control patients.

### Vaccine Effectiveness

Among patients with ILI, adjusted VE against influenza-associated outpatient illness with any influenza virus was 50.4% (Table 2). Adjusted VE against any influenza A virus subtype was 45.4%, against influenza A(H1N1)pdm09 virus was 53.3%, and against any influenza B virus was 62.3%. Among only patients in the priority vaccination groups, adjusted VE against influenza-associated outpatient illness with any influenza virus was 51.8%. Data were insufficient to estimate type- or subtype-specific VE for the priority vaccination groups.

**TABLE 2:** Interim 2025 Southern Hemisphere seasonal influenza vaccine effectiveness against influenza in patients with influenza-like illness and patients with severe acute respiratory infection — eight Southern Hemisphere countries,* March–September 2025

Influenza type, priority vaccination group,^†^ and country	Case-patients with positive influenza test results^§^		Control patients with negative influenza test results		Vaccine effectiveness^¶^	
	Total, no.	Vaccinated, no. (%)	Total, no.	Vaccinated, no. (%)	Unadjusted % (95% CI)	Adjusted % (95% CI)
**Patients with ILI **						
Any influenza virus, type A or B, total	563	78 (13.9)	1,559	375 (24.1)	49.2 (33.8 to 61.1)	50.4 (33.2 to 63.2)
Priority vaccination groups	226	49 (21.7)	841	263 (31.3)	39.2 (13.8 to 57.1)	51.8 (27.9 to 67.7)
Young children	29	1 (3.4)	218	50 (22.9)	NC	NC
Persons with comorbidities	140	23 (16.4)	350	87 (24.9)	NC	NC
Older adults	57	25 (43.9)	273	126 (46.2)	NC	NC
Influenza virus type A, total	464	65 (14.0)	1559	375 (24.1)	48.6 (31.5 to 61.4)	45.4 (24.4 to 60.5)
Priority vaccination groups	195	46 (23.6)	841	263 (31.3)	32.2 (2.6 to 52.7)	45.7 (17.5 to 64.3)
Young children	21	1 (4.8)	218	50 (22.9)	NC	NC
Persons with comorbidities	117	20 (17.1)	350	87 (24.9)	NC	NC
Older adults	57	25 (43.9)	273	126 (46.2)	NC	NC
Influenza A(H1N1)pdm09 virus, total	185	41 (22.2)	1559	375 (24.1)	10.1 (–29.6 to 37.6)	53.3 (29.3 to 69.1)
Priority vaccination groups	105	30 (28.6)	841	263 (31.3)	12.1 (–37.6 to 43.8)	55.5 (25.4 to 73.5)
Young children	17	0 (—)	218	50 (22.9)	NC	NC
Persons with comorbidities	54	13 (24.1)	350	87 (24.9)	NC	NC
Older adults	34	17 (50.0)	273	126 (46.2)	NC	NC
Influenza A(H3N2) virus, total	211	6 (2.8)	1,559	375 (24.1)	NR	NR
Priority vaccination groups	54	4 (7.4)	841	263 (31.3)	NR	NR
Young children	0	0 (—)	218	50 (22.9)	NC	NC
Persons with comorbidities	46	4 (8.7)	350	87 (24.9)	NC	NC
Older adults	8	0 (—)	273	126 (46.2)	NC	NC
Influenza virus type B, total	99	13 (13.1)	1,559	375 (24.1)	52.3 (13.5 to 73.7)	62.3 (28.8 to 80.0)
Priority vaccination groups	31	3 (9.7)	841	263 (31.3)	76.5 (21.9 to 92.9)	77.7 (19.7 to 93.8)
Young children	8	0 (—)	218	50 (22.9)	NC	NC
Persons with comorbidities	23	3 (13.0)	350	87 (24.9)	NC	NC
Older adults	0	0 (—)	273	126 (46.2)	NC	NC
Any influenza virus type A or B, by country						
Australia	354	72 (20.3)	1,088	356 (32.7)	47.5 (30.0 to 60.6)	59 (43.6 to 70.2)
New Zealand	—	—	—	—	—	—
South Africa	209	6 (2.9)	471	19 (4.0)	NR	NR
REVELAC-i	—	—	—	—	—	—
Argentina	—	—	—	—	—	—
Brazil	—	—	—	—	—	—
Chile	—	—	—	—	—	—
Paraguay	—	—	—	—	—	—
Uruguay	—	—	—	—	—	—
**Patients with SARI**						
Any influenza virus, type A or B, total	17,787	2,433 (13.7)	24,965	4,348 (17.4)	24.9 (20.7 to 28.8)	49.7 (46.3 to 52.8)
Priority vaccination groups	14,700	2,278 (15.5)	21,755	4,088 (18.8)	20.7 (16.2 to 25.1)	45.7 (41.8 to 49.3)
Young children	3,319	295 (8.9)	13,107	2,196 (16.8)	51.5 (44.9 to 57.4)	51.3 (44.5 to 57.3)
Persons with comorbidities	3,392	302 (8.9)	3,674	478 (13.0)	34.7 (23.9 to 43.9)	51.9 (43.2 to 59.3)
Older adults	7,989	1,681 (21.0)	4,974	1,414 (28.4)	32.9 (27.2 to 38.2)	37.7 (31.7 to 43.1)
Influenza virus type A, total	16,885	2,366 (14.0)	24,965	4,348 (17.4)	22.7 (18.4 to 26.8)	46.1 (42.4 to 49.6)
Priority vaccination groups	14,206	2,223 (15.6)	21,755	4,088 (18.8)	19.8 (15.2 to 24.2)	43.4 (39.3 to 47.2)
Young children	3,137	274 (8.7)	13,107	2,196 (16.8)	52.4 (45.7 to 58.3)	51.1 (44.0 to 57.3)
Persons with comorbidities	3,172	286 (9.0)	3674	478 (13.0)	33.7 (22.6 to 43.2)	48.9 (39.4 to 56.9)
Older adults	7,897	1,663 (21.1)	4974	1,414 (28.4)	32.8 (27.1 to 38.1)	35.0 (28.7 to 40.7)
Influenza A(H1N1)pdm09 virus, total	9,914	1,332 (13.4)	24,965	4,348 (17.4)	26.4 (21.4 to 31.1)	41.6 (36.7 to 46.0)
Priority vaccination groups	8,405	1,261 (15.0)	21,755	4,088 (18.8)	23.7 (18.3 to 28.8)	38.8 (33.5 to 43.8)
Young children	1,657	123 (7.4)	13,107	2,196 (16.8)	60.2 (51.9 to 67.0)	53.4 (43.5 to 61.6)
Persons with comorbidities	1,875	161 (8.6)	3,674	478 (13.0)	37.2 (24.2 to 48.0)	44.6 (31.9 to 54.9)
Older adults	4,873	977 (20.0)	4,974	1,414 (28.4)	36.9 (30.7 to 42.5)	29.7 (21.9 to 36.7)
Influenza A(H3N2) virus, total	4,490	610 (13.6)	24,965	4,348 (17.4)	25.5 (18.3 to 32.0)	37.2 (29.7 to 43.9)
Priority vaccination groups	3,822	573 (15.0)	21,755	4,088 (18.8)	23.8 (16.2 to 30.7)	34.7 (26.5 to 42.0)
Young children	913	101 (11.1)	13,107	2,196 (16.8)	38.2 (23.6 to 50.0)	30.3 (13.3 to 43.9)
Persons with comorbidities	744	43 (5.8)	3,674	478 (13.0)	59.0 (43.4 to 70.3)	58.4 (40.4 to 70.9)
Older adults	2,165	429 (19.8)	4,974	1,414 (28.4)	37.8 (29.7 to 44.9)	28.8 (17.4 to 38.6)
Influenza virus type B, total	837	56 (6.7)	24,965	4,348 (17.4)	66.0 (55.3 to 74.1)	77.6 (70.0 to 83.3)
Priority vaccination groups	445	45 (10.1)	21,755	4,088 (18.8)	51.4 (33.7 to 64.3)	74.8 (64.9 to 81.9)
Young children	172	17 (9.9)	13,107	2,196 (16.8)	45.5 (9.9 to 67.0)	64.4 (40.6 to 78.7)
Persons with comorbidities	196	15 (7.7)	3,674	478 (13.0)	44.6 (5.4 to 67.6)	71.8 (50.0 to 84.1)
Older adults	77	13 (16.9)	4,974	1,414 (28.4)	48.9 (6.9 to 71.9)	82.3 (67.1 to 90.4)
Any influenza virus type A or B, by country						
Australia	2,181	448 (20.5)	2,318	657 (28.3)	34.6 (25.0 to 43.0)	55.0 (47.0 to 61.8)
New Zealand	441	64 (14.5)	894	194 (21.7)	38.7 (16.6 to 55.0)	45.5 (22.6 to 61.7)
South Africa	—	—	—	—	—	—
REVELAC-i	15,165	1,921 (12.7)	21,753	3,497 (16.1)	24.3 (19.6 to 28.7)	40.3 (35.7 to 44.5)
Argentina	569	54 (9.5)	1,459	236 (16.2)	45.7 (25.7 to 60.3)	51.1 (32.3 to 64.7)
Brazil	13,264	1,645 (12.4)	15,698	2,435 (15.5)	22.9 (17.5 to 27.9)	40.1 (34.8 to 45.0)
Chile	607	198 (32.6)	1,679	700 (41.7)	32.3 (17.7 to 44.3)	40.5 (25.8 to 52.2)
Paraguay	634	12 (1.9)	2,680	84 (3.1)	40.4 (–9.9 to 67.6)	47.1 (2.0 to 71.5)
Uruguay	91	12 (13.2)	237	42 (17.7)	29.5 (–41.0 to 64.7)	31.9 (–41.6 to 67.2)

**Abbreviations**: ILI = influenza-like illness; NC = not calculated; NR = not reported; REVELAC-i = Pan American Health Organization Network for the Evaluation of Vaccine Effectiveness in Latin America and the Caribbean–influenza (Red para la Evaluación de Vacunas en Latino América y el Caribe–influenza); SARI = severe acute respiratory infection.

* Argentina, Australia, Brazil, Chile, New Zealand, Paraguay, South Africa, and Uruguay.

^†^ Priority vaccination groups are included as mutually exclusive groups of persons considered at high risk for severe outcomes associated with influenza infection. Young children were defined as follows, by country and age: Argentina = 6 months–1 year; Australia and Uruguay = 6 months–4 years; Brazil = 6 months–5 years; Paraguay = 6 months–2 years, Chile = 6 months–10 years; and South Africa and New Zealand = not applicable (young children not included as a priority group). Older adults were defined as those aged ≥60 years (Brazil, Chile, and Paraguay) and aged ≥65 years (Argentina, Australia, New Zealand, South Africa, and Uruguay). The preexisting conditions considered by all eight countries include chronic respiratory disease (including asthma and chronic obstructive pulmonary disease), cancer, cardiovascular disease (including hypertension and stroke), diabetes and chronic renal disease, and immunocompromising conditions (including HIV/AIDS). Some conditions were considered by select countries including obesity (Argentina, Australia, Brazil, Chile, Paraguay and Uruguay), chronic neurologic diseases (Australia and New Zealand), chronic liver disease (Australia and New Zealand), tuberculosis (New Zealand and South Africa), current alcohol or drug dependency (New Zealand), chronic hematologic disorder (Australia and New Zealand), chronic metabolic disorder (Australia), and long-term aspirin therapy in children aged 5–19 years (Australia).

^§^ Reverse-transcription polymerase–chain reaction testing for influenza was conducted at national reference laboratories.

^¶^ Vaccine effectiveness estimated from logistic regression model adjusting for sex, age in years (fit as a cubic spline with five knots), week of symptom onset (fit within each country as a cubic spline with five knots), and country.

Among patients with SARI, adjusted VE against influenza-associated hospitalization with any influenza virus was 49.7%. Adjusted VE was 46.1% against any influenza A virus subtype; adjusted VE was 41.6% against influenza A(H1N1)pdm09 and 37.2% against influenza A(H3N2). Adjusted VE against influenza B viruses was 77.6%. Among patients in the selected vaccination groups, adjusted VE against influenza-associated hospitalization with any influenza virus was 45.7%; VE was 51.3% among young children, 51.9% among persons with comorbidities, and 37.7% among older adults.

### Genetic Characterization of Viruses Reported

As of September 5, 2025, the majority of A(H1N1)pdm09 influenza viruses reported by study countries to GISAID were clade 5a.2a.1 (94.5%). Among A(H3N2) viruses, 100% were clade 2a.3a.1; 100% of influenza B viruses were the Victoria lineage and clade V1A.3a.2 (JournalofOpenSourceSoftware:Nextclade).

## Discussion

Findings from this evaluation suggest that the 2025 seasonal influenza vaccines reduced influenza-associated outpatient visits and hospitalization by an estimated one half in eight Southern Hemisphere countries. These estimates are similar to interim VE estimates from the 2024–25 Northern Hemisphere season against illness from any influenza virus in an outpatient (40%–56%) (*3*,*7*) and hospital (34%–52%) setting (*7*). Within the prioritized vaccination groups, VE against influenza A virus–associated and influenza A(H1N1)pdm09 virus–associated hospitalizations was higher among young children than among older adults, consistent with interim VE estimates from past Southern and Northern Hemisphere seasons (*3*,*4*).

Influenza vaccination provides important protectionfrominfluenzaillnessandassociatedpotentialcomplications. Despite this, 21% of patients with ILI and 16% of patients with SARI in this population had received the 2025 influenza vaccine. Surveys regarding influenza vaccine knowledge, attitudes, and practice might help to identify improved vaccine messaging and campaign approaches for increasing coverage in subsequent Southern Hemisphere seasons.

Examination of seasonal influenza VE in the Southern Hemisphere can provide information for influenza vaccine composition deliberations for the subsequent Southern Hemisphere season. In addition, these VE estimates help to prepare Northern Hemisphere health authorities for anticipated levels of protection that influenza vaccines might provide, should similar viral clades predominate during the 2025–26 season (*8*). To add to mitigation efforts against severe illness in the coming season, health care providers can recommend the use of antivirals, where available, for patients with suspected or confirmed influenza.

### Limitations

The findings in this report are subject to at least six limitations. First, the interim VE estimates included are preliminary and might differ from end-of-season estimates. Second, estimates for patients with ILI were generated using a small analytic sample which reduced precision and prevented estimation of VE across all subgroups. Third, despite use of high-quality surveillance data, 61% of patients were excluded because of missing RT-PCR results, which might have biased estimates and suggests a need to strengthen the integration of laboratory and epidemiologic data used to support this analysis. Fourth, this analysis was unable to distinguish between previously unvaccinated young children who received 1 dose versus the recommended 2 doses of influenza vaccine, potentially biasing VE among this population. Fifth, the sequenced specimens reported to GISAID are not necessarily the same as those from patients included in this VE evaluation. Finally, these VE estimates might not be generalizable to Southern Hemisphere countries that have had different circulating viruses in the 2025 season.

### Implications for Public Health Practice

Interim VE estimates for the Southern Hemisphere 2025 influenza season suggest that influenza vaccines were effective in reducing influenza-associated outpatient visits and hospitalization by approximately one half. Examination of influenza VE during the Southern Hemisphere season might provide insights for health authorities who are actively preparing and planning for the upcoming Northern Hemisphere influenza season. The 2025–2026 Northern Hemisphere seasonal influenza vaccine composition is the same as the 2025 Southern Hemisphere seasonal influenza vaccine; health authorities in Northern Hemisphere locations might anticipate similar levels of protection against influenza illness, should the same influenza viruses circulate during the upcoming season. These findings support CDC’s recommendations for all eligible persons aged ≥6 months to receive a seasonal influenza vaccine before the start of the Northern Hemisphere influenza season (*9*).

## Footnotes

* These authors contributed equally to this report.

† ILI is defined as acute respiratory illness (ARI) with history of fever or documented body temperature of ≥100.4°F (≥38°C), cough and symptom onset within the previous 10 days (South Africa); and ARI with fever and cough with onset of symptoms within the previous 7 days (Australia). SARI is defined as history of fever or documented body temperature of ≥100.4°F (≥38°C) and cough with onset within the previous 10 days resulting in hospitalization​ (REVELAC-i countries and New Zealand); hospital admission with ARI (age >16 years: ARI symptoms; age <16 years: ARI symptoms or fever) (Australia).

§ Data were not contributed from the North Region of Brazil, which uses the Northern Hemisphere vaccine formulation.

¶ Influenza vaccination campaign start dates were March 1 (Chile), March 25 (Argentina), March 27 (Australia), March 31 (South Africa and Uruguay), April 1 (New Zealand), April 3 (Paraguay), and April 7 (Brazil).

** Trivalent vaccines containing antigens from A/Victoria/4897/2022 (H1N1)pdm09–like virus, A/Croatia/10136RV/2023 (H3N2)–like virus, and B/Austria/1359417/2021 (B/Victoria lineage)–like virus were used in Argentina, Brazil, Chile, Paraguay, South Africa, and Uruguay; quadrivalent vaccines containing the addition of a B/Yamagata lineage–like virus were used in Australia, New Zealand, South Africa, and Uruguay; adjuvanted vaccines were available for older adults in Argentina (aged ≥65 years), Australia (aged ≥65 years), and Paraguay (aged ≥60 years).

†† VE was estimated using multivariable logistic regression as (1 − adjusted odds ratio) × 100%.

§§ Young children aged 6 months–1 year (Argentina), 6 months–4 years (Australia and Uruguay), 6 months–5 years (Brazil), 6 months–2 years (Paraguay), and 6 months–10 years (Chile). In South Africa and New Zealand, young children are not considered a priority vaccination group.

¶¶ Comorbid conditions included chronic respiratory disease (including asthma and chronic obstructive pulmonary disease); cancer; cardiovascular disease (including hypertension and stroke); diabetes/chronic renal disease; and immunocompromising conditions (including HIV/AIDS) in all countries. In selected countries, the following comorbid conditions were included: obesity (Argentina, Australia, Brazil, Chile, Paraguay, and Uruguay); chronic neurologic diseases (Australia and New Zealand); chronic liver disease (Australia and New Zealand); tuberculosis (New Zealand and South Africa); current alcohol or drug dependency (New Zealand); chronic hematologic disorder (Australia and New Zealand); chronic metabolic disorder (Australia); and long-term aspirin therapy in children aged 5–19 years (Australia).

*** Aged ≥60 years (Brazil, Chile, and Paraguay) and aged ≥65 years (Argentina, Australia, New Zealand. South Africa, and Uruguay).

††† 45 C.F.R. part 46. 102(1)(2), 21 C.F.R. part 56; 42 U.S.C. Sect. 241(d); 5 U.S.C. Sect. 552a; 44 U.S.C. Sect. 3501 et seq.
